# Relationship between exercise motivation and social support in a support facility for persons with disabilities in Japan

**DOI:** 10.1080/07853890.2022.2049860

**Published:** 2022-03-14

**Authors:** Yasuhiro Inui, Yoichi Tanaka, Tatsuya Ogawa, Kazuki Hayashida, Shu Morioka

**Affiliations:** a Department of Neurorehabilitation, Graduate School of Health Sciences, Kio University, Nara, Japan; b Nara Prefecture General Support Center for Persons with Disabilities, Nara, Japan; c Department of Rehabilitation, Nara Prefecture General Rehabilitation Center, Nara, Japan; d Department of Rehabilitation Medicine, Nishiyamato Rehabilitation Hospital, Nara, Japan; e Department of Rehabilitation, Fujiikai Rehabilitation Hospital, Osaka, Japan; f Neuro rehabilitation Research Center, Kio University, Nara, Japan

**Keywords:** Support facility, persons with disabilities, exercise motivation, social support, family support, individual capabilities

## Abstract

**Purpose:**

Exercise motivation (EM) is related to individual capabilities and social support. However, in support facilities for people with disabilities, it is susceptible to a lack of social support. In this study, we classified EM into Autonomous Motivation (AM) and controlled motivation (CM) and then examined the influence of social support.

**Method:**

Thirty-three residents from a support facility for people with disabilities in Japan participated in this study. We conducted a hierarchical multiple regression analysis in which age, gender and time since admission were entered in Step 1, mobility and self-efficacy as individual capabilities in Step 2, and family support, facility support and peer support as social support in Step 3.

**Result:**

A significant increase in variance from Step 2 to Step 3 was found for both AM (Δ*R*^2^ = 0.504, Δ*F* = 12.18, *p* < .001) and CM (Δ*R*^2^ = 0.269, Δ*F* = 3.491, *p* = .031). The results also showed that AM was higher among those with high family and facility support, and CM was higher among those with low family and high peer support.

**Conclusions:**

Social support was a more significant predictor of EM among participants than individual capabilities.

**KEY MESSAGES**
- Among residents of support facilities for people with disabilities, assessing not only individual capabilities but also social support status can lead to better understandings of exercise motivation (EM).
- To enhance facility residents’ autonomous motivation (AM), it is necessary to intervene after evaluating family and facility support.
- When family support is not readily available among facility residents, efforts should be made to encourage residents to interact with each other to increase peer support.

## Introduction

According to the World Health Organization, over 1 billion people were estimated to live with some form of disability globally in 2020, and the lack of health care services for the disabled was seen as a growing problem [1]. The Japanese Cabinet Office reports that, in 2020, there were 4.12 million physically disabled people over the age of 18 in Japan, and 70,000 of them were in institutions [2]. Facilities where people with disabilities stay for a long time are called long-term care facilities (LTCFs), they vary in form from country to country and there are many types in each country [3]. In Japan, one type of LTCF is support facilities for people with disabilities. In these facilities, “residential support” is provided to persons with disabilities throughout the night to early morning and daily life care self-support training, support for transition to employment and support for continuous employment are provided during the daytime [4]. Facility residents are limited to people with disabilities under the age of 65 and they are required to engage in exercise to maintain and improve their physical functions to realize social participation. The standard duration of institutionalization is 18 months [5] and requires a long period of continuous exercise. It is important for exercise motivation (EM) to be high to encourage long-term persistence [6] and maximize improvements in physical function [7] towards social involvement. Understanding the factors related to EM in LTCFs will contribute to the development of effective intervention strategies that will inspire continuous exercise among residents.

### Factors associated with exercise motivation

EM has been studied among inpatients in rehabilitation hospitals and is an important determinant of rehabilitation outcomes [7]. During the rehabilitation period, EM is said to be influenced by individual capabilities such as mobility [8] and self-efficacy [9,10]. On the other hand, it has been reported that social support such as family support [11,12], facility support [13,14] and peer support [15] affects EM. Thus, EM among inpatients in rehabilitation hospitals is influenced by both individual capabilities and social support.

### Social support in a support facility for people with disabilities

Patients who are admitted to a rehabilitation hospital aim to improve their mobility. Thus, they engage in exercise with the full support of doctors, nurses and physical therapists, and spend time with patients of various ages and with various conditions. On the other hand, residents of LTCFs are required to engage in exercise under different circumstances than those in hospitals. The Ministry of Health, Labour and Welfare (MHLW) in Japan reports that about 40% of admissions in LTCFs are for the purpose of acquiring mobility and living skills, while 50% are for social reasons, such as the lack of adequate support at home [16]. Therefore, LTCF residents may struggle to receive sufficient family support. Additionally, the MHLW reported problems such as an insufficient number of staff and difficulties in providing individualized support in the facilities [5]. Therefore, it is possible that the LTCF residents may not be fully satisfied with their facility support. The MHLW also reported that most facility residents were between 40 and 60 years old, and more than half of them were patients with cerebrovascular diseases [5]. Since patients of the same age and with the same condition often live together for longer periods, it is thought that peer support is easier to obtain among LTCF residents. Based on these characteristics of social support, and the association mentioned previously between EM and social support, some residents may be less motivated to exercise due to low family and facility support, while others may be more motivated due to high peer support. Consequently, social support may play a unique role in predicting EM over and above individual capabilities, such as mobility and self-efficacy among LTCF residents. However, there are no published studies that have examined this topic in detail.

### Autonomous motivation and controlled motivation

Previous studies that have examined factors related to EM in rehabilitation have focussed on whether motivation is “high” or “low,” and there are few reports that have examined motivation itself in a categorized manner. On the other hand, self-determination theory [17], a major motivation theory developed in recent years, proposes classifying motivation into autonomous motivation (AM), which is motivated by one’s own volition and decisions and controlled motivation (CM), which is motivated by external incentives [18]. AM and CM are considered to be independent constructs, and it is necessary to consider motivation from both aspects [19]. In the rehabilitation discourse, it has been reported that AM is involved in the amount of exercise that patients recovering from strokes partake in after discharge from the hospital [20], while the necessity of CM has been reported in inpatient rehabilitation [21] and outpatient rehabilitation for chronic obstructive pulmonary disease patients [22]. Furthermore, CM has been reported to be enhanced by ambient support (such as nurses) in the self-management behaviour of dialysis patients [23]. Considering that support facilities for persons with disabilities, which are this study’s target, need to support LTCF residents in their efforts to exercise and achieve social participation, it is necessary to focus on CM. Therefore, considering EM from the aspects of AM and CM will lead to a better understanding of EM among LTCF residents.

### Study purpose

Social support and individual capability are associated with EM. Considering the social support problems and characteristics in support facilities for persons with disabilities, it is assumed that social support is strongly related to EM. Furthermore, we thought that considering EM from the perspective of CM as well as AM would help capture its characteristics among LTCF residents. Therefore, this study’s purpose was to quantitatively investigate EM in LTCF residents in terms of AM and CM, and to examine the influence of social support after controlling for individual capabilities.

## Methods

### Participants

In this study, the residents of Nara Prefecture General Support Centre for Persons with Disabilities were recruited as participants. About 15 residents are admitted to this facility each year. They are required to participate in exercise programmes, such as group training on mats and group gait training in five 90-min sessions each week. Additionally, some residents participate in physical therapy and occupational therapy programmes for 20 min once or twice a week, and cognitive function training and social adaptation training at other times. The inclusion criteria were residents who agreed to participate in the study. Exclusion criteria included those who had been in the facility for less than 3 months, had aphasia, cognitive impairment (mini-mental state examination ≦ 21 points) [24] or psychiatric disorders.

### Measures

Data regarding demographic characteristics were gathered including age, gender, time since admission and other health conditions. Furthermore, several measures were included to assess the study variables.

### Exercise motivation

EM was assessed using a 19-item Behavioural Regulation in Exercise Questionnaire (BREQ-2) [25]. The scale comprises five subscales: amotivation with 4 items (e.g. “I don’t see why I should have to exercise”), external regulation with 4 items (e.g. “I exercise because other people say I should”), introjected regulation with three items (e.g. “I feel guilty when I don’t exercise”), identified regulation with four items (e.g. “I value the benefits of exercise”), and intrinsic motivation with four items (e.g. “I exercise because it’s fun”). The items were scored on a 5-point Likert scale, ranging from 0 (Not true for me) to 4 (Very true for me). The BREQ2 has been validated for reliability and validity [25] and, more recently, has been shown to be reliable and valid for exercise in elderly care facilities [26]. The BREQ-2 subscales “identified regulation” and “intrinsic regulation” were merged into the variable AM, and “external regulation” and “introjected regulation” were merged into the variable CM [27]. Mean item scores are reported for each subscale. The mean item score for each item was used as the AM and CM values. It has also been reported that classification into AM and CM rather than subscales improves validity [28]. In this study, the “exercise” to be assessed using the BREQ-2 was set as a group mat exercise and walking practice programme, the main exercise sessions in the facility. The participants were asked to fill in a questionnaire after their exercise sessions.

### Mobility

Mobility was assessed using the Rivermead Mobility Index (RMI), which is a 15-item (15-point) rating scale consisting of 14 questions and one behavioural observation item and has been reported to be highly valid and reliable [29]. Maeshima developed a Japanese version of the RMI and its reliability and validity have been verified [30]. In this study, we evaluated mobility using the Japanese version.

### Self-efficacy

Self-efficacy was assessed using the general self-efficacy scale (GSES). Self-efficacy refers to the degree of confidence that an individual has in his or her ability to carry out an action in a variety of different and difficult situations. The GSES questionnaire’s reliability and validity have been proven by Sakano and Tohjoh [31]. The total score ranges from 0 to 16 points, with lower scores indicating lower self-efficacy in performing daily activities.

### Social support

With regard to social support, we investigated perceived social support (how an individual feels about the social support they receive), as it has been addressed in previous research on motivation in rehabilitation [7]. For family support, we used an adapted version of Zimet et al.’s [32]. Multidimensional Scale of Perceived Social Support (MSPSS). This scale was adapted by Iwasa in Japanese [33], and its reliability and validity have been verified. This instrument consists of 12 items and examines one’s subjective perception of the social support at one’s disposal from three sources: family, friends and significant others (excluding family). The respondents were asked to indicate how they feel about each statement presented to them. Responses were given on a 7-point scale, from 1 (very strongly disagree) to 7 (very strongly agree). In this study, items were selected and adopted from the MSPSS to fit the purpose of the study. Since one of the objectives of this study was to investigate family support, support from friends was not measured. Additionally, because some residents did not have immediate family members, we considered support from significant others (excluding family) to be equivalent to support from family members. Therefore, we measured family support for all subjects using a scale that combined support from significant others and support from family members. Facility support was evaluated using SERVQUAL [34], a scale that evaluates service quality using perceived quality (how, whether, and to what degree a service is perceived as valuable by the recipient). SEVQUAL has been used across various industries and is also used to evaluate hospital services. In Japan, Nakamura [35] devised a rating scale with a total of six dimensions (18 items), adding “technicality” to the original method (tangibles, reliability, responsiveness, assurance and empathy). The scoring method is based on a 7-point scale, ranging from 1 (completely disagree) to 7 (strongly agree). This is a rating scale for service satisfaction, but we decided to treat it as facility support because the questions were equivalent or at least similar to support perception. Peer support was assessed using Ono’s Peer Support Scale (OPSS) [36]. This scale consists of 20 items in three factors: 10 items for “providing support,” 6 items for “accepting emotional support” and 4 items for “accepting informational support.” The scale is rated on a 4-point scale from 3 (often) to 0 (rarely); the higher the score, the better the peer support function.

### Procedure

The ethical approval for this study was received from the ethics committee of the Nara Prefecture General Rehabilitation Center (R1-2). Data collection for this study began in October 2019 and ended in December 2020. No pre-test power analysis was conducted because no previous studies have investigated the factors associated with EM in support facilities for persons with disabilities. This study was conducted in compliance with the Declaration of Helsinki, and sufficient care was taken to ensure that personal information was not revealed. Participants were briefed on the evaluation and completed the questionnaire by themselves in the presence of a research assistant. All participants signed an informed consent form before answering the questionnaire.

### Data analysis

Descriptive statistics analysis and Cronbach’s alphas were conducted for sample characteristics and subscale reliabilities. Since a Cronbach’s alpha of 0.7 or higher is considered appropriate and less than 0.5 is unacceptable [37], we judged higher than 0.5 to be reliable. The normal distributions of all data were tested by the Shapiro–Wilk test. As a result, parametric tests were performed for those with normality in the data distribution, and nonparametric tests were performed for those without normality. To examine correlations among the measures, Pearson’s correlation coefficient was conducted for AM and GSES, AM and SERVQUAL, AM and OPSS, GSES and SERVQUAL, GSES and OPSS and SERVQUAL and OPSS, while Spearman’s rank correlation coefficient was conducted for the rest. A three-step hierarchical multiple regression using the forced entry method was performed to predict AM and CM. First, the demographics were entered (age, gender and time since admission), followed by individual capabilities variables (mobility (RAI) and self-efficacy (GSES)), and finally social support variables (family (MSPSS), facility (SERVQUAL) and peer (OPSS) support). The *F*-test was used to determine the significance of the multiple regression model. The multicollinearity assumption was rejected, with the maximal VIF measure of predictors being 1.31. A *post-hoc* power calculation was performed to determine the power using the computer program G*Power version 3.1.7, after data collection. The power was calculated from the sample and the effect sizes, with significance set at 5%. A complete case analysis was performed, and the missing values were excluded. HAD version 16.057 [38] was used for the statistical analysis and the statistical significance level was set at 5%.

## Results

**Figure 1.**
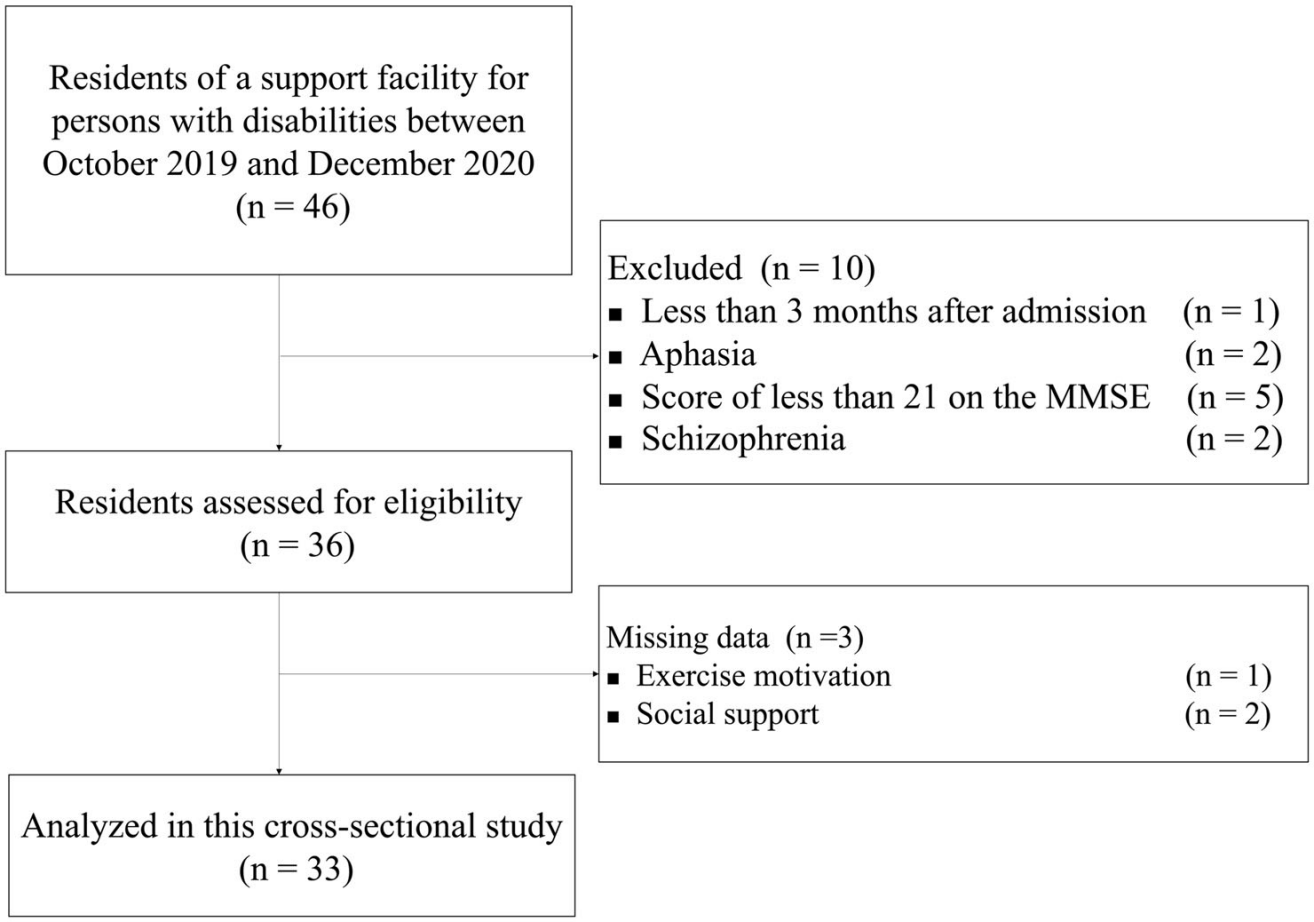
Participant inclusion criteria flow diagram.

Figure 1 presents the flow diagram of participants who were considered and recruited for this study. A total of 46 residents were admitted to the persons with disabilities facility to which the researcher is affiliated, between October 2019 and December 2020. Ten residents were excluded because they did not meet the inclusion criteria; consequently, 36 residents were enrolled in this study. Three residents did not complete the questionnaires by either refusing to answer or citing difficulties in understanding the questions. Finally, data from 33 residents were analysed.

The demographic details and characteristics of the injuries of the participants are presented in Table 1. The median age of the participants was 55.0 (IQR 16.0), and the majority of participants were male (*n* = 27, 81.8%). The median time since admission was 7.0 months (IQR 17.0); 84.8% of the participants had an acquired brain injury, 9.1% had spinal cord injury, 3.0% had polyneuritis and 3.0% had amputated limbs.

**Table 1.** Participant demographic characteristics and injury characteristics (*n* = 33).

Variable	Median (IQR)	*n* (%)	Range
Age (years)	55.0 (16.0)	–	23–74
Time since admission (month)	7.0 (17.0)	–	3–46
Gender	–		–
Female	–	6 (18.2%)	–
Male	–	27 (81.8%)	–
Condition	–		–
Acquired brain injury	–	28 (84.8%)	–
Spinal cord injury	–	3 (9.1%)	–
Polyneuritis	–	1 (3.0%)	–
Amputee	–	1 (3.0%)	–

IQR: inter quartile range.

Descriptive data for the variables measured in this study can be found in Table 2. The internal consistency of each scale is also provided in this table. AM, GSES, MSPSS and OPSS were found to be highly reliable with Cronbach’s *α* of 0.823, 0.747, 0.960, 0.959 and 0.955, respectively. There was adequate internal consistency for CM with Cronbach’s *α* of 0.682. Thus, the measures used had the necessary reliability for the study.

**Table 2.** Descriptive values for study variables (*n* = 33).

Variable	Score	Range	Cronbach’s *α*
AM^a^	2.7 ± 0.8	1.1–4.0	0.823
CM^b^	0.9 (1.0)	0–3.1	0.682
RMI^b^	8.0 (7.0)	1–15	NA
GSES^a^	8.7 ± 3.5	3–14	0.747
MSPSS^b^	47.0 (16.0)	4–56	0.960
SERVQUAL^a^	83.6 ± 20.2	29–120	0.959
Ono’s Peer support scale^a^	26.1 ± 15.0	0–51	0.955

AM: autonomous motivation; CM: controlled motivation; RMI: Rivermead Mobility Index; GSES: General Self-Efficacy Scale; MSPSS: Multidimensional Scale of Perceived Social Support.

^a^Mean ± standard deviation; ^b^median (inter quartile range).

### Correlations among variables

A correlational matrix showing relationships across all variables is presented in Table 3. There were significant correlations between several of the variables. AM had a strong positive relationship with gender (*r* = 0.409, *p* = .018), family support as measured by the MSPSS (*r* = 0.544, *p* = .001), and facility support as measured by the SERVQUAL (*r* = 0.534, *p* = .001). CM had a moderate positive relationship with OPSS (*r* = 0.371, *p* = .033). However, there was no significant correlation for both RMI and GSES with either AM or CM.

**Table 3.** Correlations of study variables.

	2	3	4	5	6	7	8	9	10
1. AM	−0.115	−0.011	0.409*	−0.034	0.029	0.048	0.544**	0.534**	0.285
2. CM	–	−0.147	0.153	−0.230	0.274	0.098	−0.298	−0.016	0.371*
3. Age		–	0.112	−0.209	0.041	−0.210	0.159	−0.016	0.007
4. Gender			–	−0.100	−0.046	0.054	0.207	0.223	0.265
5. TSA				–	−0.036	0.150	−0.266	0.068	−0.093
6. RMI					–	0.068	−0.109	0.056	0.184
7. GSES						–	0.260	−0.071	0.178
8. MSPSS							–	0.169	0.138
9. SERVQUAL								–	−0.056
10. OPSS									–

AM: autonomous motivation; CM: controlled motivation; TSA: time since admission; RMI: Rivermead Mobility Index; GSES: General Self-Efficacy Scale; MSPSS: Multidimensional Scale of Perceived Social Support; OPSS: Ono’s Peer support scale.

*p < .05; **p < .01.

### Hierarchical regression analysis

The results of the hierarchical regression analysis examining how demographic contexts, individual capabilities and social support relate to EM are presented in Tables 4 and 5. For AM, in Step 2, the variables for individual capabilities (mobility and self-efficacy) were entered and did not demonstrate any significant contribution to the variance (*R*^2^ = 0.165, adjusted *R*^2^ = 0.010, *F* (5, 27) = 1.07, *p* = .401). In Step 3, social support variables (MSPSS, SERVQUAL and OPSS) were entered to assess their contribution to the model, over and above the variables for demographic contexts and individual capabilities. The addition of these variables contributed to a 50.4% increase in the amount of AM variance explained (Δ*R*^2^ = 0.504, Δ*F* = 12.18, *p* < .001). The final overall model had an *R*^2^ of 0.669 and an adjusted *R*^2^ of 0.558 (*F* (8, 24) = 6.06, *p* < .001), indicating that approximately half of the variance in AM could be explained by the variables entered into the model. The significant independently contributing predictors of AM were high MSPSS (*β* = 0.550, *p* < .001) and high SERVQUAL (*β* = 0.365, *p* = .009) (see Table 4). For CM, the variables did not demonstrate any significant contribution to the variance in both Step 2 (*R*^2^ = 0.114, adjust *R*^2^ = −0.050, *p* = .630) and Step 3 (*R*^2^ = 0.384, adjust *R*^2^ = 0.178, *p* = .113), separately. From Steps 2 to 3, however, the addition of these variables contributed to a 25.9% increase in the amount of CM variance (Δ*R*^2^ = 0269, Δ*F* = 3.491, *p* = .031). The significant independent contributing predictors of CM were low MSPSS (*β* = −0.503, *p* = .012) and high OPSS (*β* = 0.365, *p* = .046) (see Table 5).

**Table 4.** Hierarchical regression analysis for predicting autonomous motivation.

Step and variables	*R* ^2^	*ΔR* ^2^	*β*	95% CI	*p*
Step 1	0.160	–	–	–	–
Age	–	–	−0.014	−0.37–0.34	.937
Gender	–	–	0.392	0.04–0.75	.031*
TSA	–	–	0.117	−0.23–0.47	.497
Step 2	0.165	0.005			
Age	–	–	−0.006	−0.38–0.37	.973
Gender	–	–	0.395	0.03–0.76	.036*
TSA	–	–	0.116	−0.25–0.48	.523
RMI	–	–	0.069	−0.29–0.43	.698
GSES	–	–	0.005	−0.37–0.38	.978
Step 3	0.669**	0.504*			
Age	–	–	−0.135	−0.40–0.13	.310
Gender	–	–	0.205	−0.06–0.47	.120
TSA	–	–	0.203	−0.05–0.46	.111
RMI	–	–	0.015	−0.23–0.26	.903
GSES	–	–	−0.160	−0.43–0.11	.237
MSPSS	–	–	0.550	0.27–0.83	.000**
SERVQUAL	–	–	0.365	0.10–0.63	.009**
OPSS	–	–	0.211	−0.05–0.47	.110

AM: autonomous motivation; CM: controlled motivation; TSA: time since admission; RMI: Rivermead Mobility Index; GSES: General Self-Efficacy Scale; MSPSS: Multidimensional Scale of Perceived Social Support; OPSS: Ono’s Peer support scale.

**p < .01; *p < .05.

**Table 5.** Hierarchical regression analysis for predicting controlled motivation.

Step and variables	*R* ^2^	Δ*R*^2^	*β*	95% CI	*p*
Step 1	0.084	–	–	–	–
Age	–	–	−0.082	−0.45–0.29	.652
Gender	–	–	0.256	−0.11–0.63	.166
TSA	–	–	−0.121	−0.49–0.24	.503
Step 2	0.114	0.030			
Age	–	–	−0.062	−0.45–0.32	.742
Gender	–	–	0.263	−0.12–0.64	.166
TSA	–	–	−0.125	−0.50–0.25	.503
RMI	–	–	0.173	−0.20–0.55	.352
GSES	–	–	0.020	−0.37–0.41	.917
Step 3	0.384	0.269*			
Age	–	–	0.068	−0.30–0.43	.704
Gender	–	–	0.209	−0.15–0.57	.241
TSA	–	–	−0.156	−0.50–0.19	.362
RMI	–	–	0.095	−0.24–0.44	.568
GSES	–	–	0.115	−0.26–0.49	.528
MSPSS	–	–	−0.503	−0.88 to −0.12	.012*
SERVQUAL	–	–	0.134	−0.23–0.50	.452
OPSS	–	–	0.365	0.01–0.72	.046*

AM: autonomous motivation; CM: controlled motivation; TSA: time since admission; RMI: Rivermead Mobility Index; GSES: General Self-Efficacy Scale; MSPSS: Multidimensional Scale of Perceived Social Support; OPSS: Ono’s Peer support scale.

*p < .05.

In the *post-hoc* power calculation for AM (effect size *f*^2^ = 1.00, total sample size = 33, number of predictors = 8 and *α* = 0.05), the power was 0.960. In the *post-hoc* power calculation for CM (effect size *f*^2^ = 0.217, total sample size = 33, number of predictors = 8 and *α* = 0.05), the power was 0.217.

## Discussion

In this study, we quantitatively investigated the EM among residents of a support facility for persons with disabilities by classifying it into AM and CM, and examined the role of social support. Hierarchical multiple regression analysis showed that both AM and CM were influenced by social support when the influence of individual capabilities was controlled for. Therefore, our hypothesis was supported. Furthermore, AM was found to be higher when family support and facility support were higher, and CM was found to be higher when family support was lower and peer support was higher; although, in the final (Step 3) model, demographics, individual capabilities and social support were entered, and did not demonstrate any significant contribution to the variance in CM.

The strong influence that social support had on EM may have been influenced by the environment of support facilities for persons with disabilities. As mentioned at the beginning of this article, in LTCF, it is generally understood that family support [16] and facility support [5] are difficult to obtain, and peer support [5] is easier to obtain. Social support has also been an issue in LTCFs in other countries [39]. So, LTCF residents need to engage in long-term exercise under these conditions. Therefore, the degree of social support may have a strong influence on their EM.

Hierarchical multiple regression analysis showed that AM was higher when family support and facility support were higher and that these two social support structures together explained more than 50% of the variance in AM (Table 4). Each of these social supports has been reported to promote autonomy in elderly people who have been in LTCFs for a long time [40,41]. Therefore, the results of this study are consistent with the results of previous studies in this context. On the other hand, it may be a feature of the residents in the support facilities for persons with disabilities that both of these types of social support affect AM. People with disabilities under 65 years of age, who are admitted to LTCFs, are usually at an age where they are engaged in work, support their families and have many social relationships. Further, if they have a disability at that age, they need not one but several social supports to maintain their psychosocial domain of health-related quality of life [42]. Additionally, when they develop a disability and are admitted to a facility, they experience a disconnect with society [39]; so, in such a situation, not only a single social support but the support of both family members who live apart from them and staff members who assist them during their stay at the facility may stabilize their psychological state and allow them to tackle their problems autonomously.

All the variables entered did not significantly predict the total variation in CM, but those with lower family support and higher peer support were shown to have higher CM. Family support is an important social support for patients, for example, it has been reported that stroke patients are less likely to see friends and acquaintances, so they are more connected to their families [43], and that family participation in rehabilitation has a positive impact on patients’ psychological and functional outcomes [44]. Therefore, when family support declines in facilities, peer support may function complementarily to the social support that has become scarce. Additionally, peer support has been reported to promote LTCF residents’ participation in recreation [45], motivation for treatment [41] and self-management behaviour [46]. Therefore, it is possible that peer support caused an increase in external motivation.

The social support structures associated with AM and CM were different, with high family support and high facility support being predictors of AM, and low family support and high peer support being predictors of CM. Since it is necessary to consider EM from both the AM and CM aspects [19], it is also necessary to consider the role of social support in EM separately. Furthermore, family support, which was associated with both AM and CM and had conflicting effects, may be important when considering EM among LTCF residents. For example, if both AM and CM are low, and family support is also low, it may be necessary to increase AM by improving the level of family support or CM by improving the level of peer support. Family support has been reported to be counterproductive to motivation when it is excessive [47]. However, considering that difficulties in receiving family support are often cited as a reason for admitting persons with disabilities to LTCFs [16], further research focussing on family support is required in the future.

Previous studies have shown that individual capabilities such as mobility [8] and self-efficacy [9,10] are associated with EM. However, in this study, individual capabilities, such as mobility and self-efficacy were not associated with EM. In the existing literature, motivation for individual ability-based exercise programmes provided in acute and subacute rehabilitation has been investigated [48]. However, in LTCFs, many participants exercise in groups rather than individually, and a uniform exercise programme is provided in a group. Therefore, people with low mobility may be able to participate with high motivation, expecting to improve their physical functions by exercising, while people with high mobility may be less challenged and less motivated in group exercise. In fact, it has been reported that LTCF residents were not provided with sufficient exercise programmes to meet their expectations [49]. Thus, high mobility may not necessarily motivate individuals to exercise. Additionally, the self-efficacy measured in this study is general self-efficacy, which is related to “being able to be proactive in appropriate problem-solving behaviors” [50] but has been reported to have little effect on routinely repeated actions [10]. Because the exercise programme in the facility was repeated uniformly in groups, participants’ EM may have been less influenced by self-efficacy. Additionally, the problem of uniform exercise programmes has also been pointed out in LTCFs across the world [3], so it is necessary to consider social support rather than individual capabilities when thinking about EM for LTCF residents.

### Limitations and future directions

The first limitation of this study is that it was conducted only in one facility. The gender, age, reason for admission and disability type of the residents were limited. These aspects, as well as the environment, are expected to vary from facility to facility. So, the results of this study may reflect the characteristics of our facility. Therefore, it is necessary to investigate the impact of environmental factors on EM in other facilities as well. Second, since there were missing data as participants struggled to answer the questionnaire, it is necessary to simplify the questionnaire when the survey is conducted in other facilities. Third, the power of the model testing the association of the independent variables with CM was low. Therefore, we do not have evidence to suggest that the characteristics of the other dimensions are not associated with CM, and further research is required. Fourth, this study was a cross-sectional study, and did not capture longitudinal changes after admission to the facility, so the causal relationships were not clear. We believe that investigating how the EM changes over time from the time of admission, and which social support is associated with it, will clarify the impact of admission to a facility, length of stay and social support on EM. In particular, the relationship between AM and social support has been clarified, but the relationship among the social support structures remains unknown. We expect that a longitudinal study will reveal the relationship between these in the future.

### Summary and clinical implications

This study supports the importance of social support in predicting EM among LTCF residents. Additionally, when EM was categorized into AM and CM, it was also found that different social support systems were associated with each of the two categories. Although previous studies have already reported that individual capabilities and social support are related to EM, this is the first study to focus on the importance of social support in a specific environment, such as a support facility for persons with disabilities. The findings of this study suggest that understanding and adjusting the way that LTCF residents interact with their families and facility staff can lead to autonomous efforts in exercising and that it is necessary to enhance peer support to promote interaction among residents to increase their external motivation. The results of this study provide a basis for further study and deeper examination of the factors that promote commitment to exercise in support facilities for persons with disabilities.

## Data Availability

The data that support are openly available in figshare at http://doi.org/10.6084/m9.figshare.16620454.
